# Different Patterns of Cytokines and Chemokines Combined with IFN-γ Production Reflect *Mycobacterium tuberculosis* Infection and Disease

**DOI:** 10.1371/journal.pone.0044944

**Published:** 2012-09-13

**Authors:** Yang Yu, Yan Zhang, Shizong Hu, Dongdong Jin, Xinchun Chen, Qi Jin, Haiying Liu

**Affiliations:** 1 MOH Key Laboratory of Systems Biology of Pathogens, Institute of Pathogen Biology, Chinese Academy of Medical Sciences & Peking Union Medical College, Beijing, China; 2 Shenzhen-Hong Kong Institute of Infectious Disease, Shenzhen Third People’s Hospital, Guangdong Medical College, Shenzhen, China; South Texas Veterans Health Care System and University Health Science Center San Antonio, United States of America

## Abstract

**Background:**

IFN-γ is presently the only soluble immunological marker used to help diagnose latent *Mycobacterium tuberculosis* (*M.tb)* infection. However, IFN-γ is not available to distinguish latent from active TB infection. Moreover, extrapulmonary tuberculosis, such as tuberculous pleurisy, cannot be properly diagnosed by IFN-γ release assay. As a result, other disease- or infection-related immunological biomarkers that would be more effective need to be screened and identified.

**Methodology:**

A panel of 41 soluble immunological molecules (17 cytokines and 24 chemokines) was tested using Luminex liquid array-based multiplexed immunoassays. Samples, including plasma and pleural effusions, from healthy donors (HD, n = 12) or patients with latent tuberculosis infection (LTBI, n = 20), pulmonary tuberculosis (TB, n = 12), tuberculous pleurisy (TP, n = 15) or lung cancer (LC, n = 15) were collected and screened for soluble markers. Peripheral blood mononuclear cells (PBMCs) and pleural fluid mononuclear cells (PFMCs) were also isolated to investigate antigen-specific immune factors.

**Principal Findings:**

For the 41 examined factors, our results indicated that three patterns were closely associated with infection and disease. (1) Significantly elevated plasma levels of IL-2, IP-10, CXCL11 and CXCL12 were present in both patients with tuberculosis and in a sub-group participant with latent tuberculosis infection who showed a higher level of IFN-γ producing cells by ELISPOT assay compared with other latently infected individuals. (2) IL-6 and IL-9 were only significantly increased in plasma from active TB patients, and the two factors were consistently highly secreted after *M.tb* antigen stimulation. (3) When patients developed tuberculous pleurisy, CCL1, CCL21 and IL-6 were specifically increased in the pleural effusions. In particular, these three factors were consistently highly secreted by pleural fluid mononuclear cells following *M.tb*-specific antigen stimulation. In conclusion, our data imply that the specific secretion of soluble immunological factors, in addition to IFN-γ, may be used to evaluate *M.tb* infection and tuberculosis disease.

## Introduction

Tuberculosis is a global infectious disease that is mainly caused by *Mycobacterium tuberculosis* (*M.tb*). Approximately 30% of the world’s population is affected by *M.tb* infection, which causes 1.7 million deaths every year. According to the 2009 WHO report on tuberculosis, there were an estimated 9.4 million incident cases of TB globally, which was equivalent to 137 cases per 100,000 people [1], [2]. Although several immunological methods are currently used to diagnose latent tuberculosis infection and support a TB diagnosis, including the tuberculin skin test or interferon-γ release assay, these assays are not conclusive and still require sophisticated technology for appropriate and effective diagnoses [3], [4], [5]. For example, it is difficult to diagnose tuberculous pleuritis [6]. Microbiological tests are still the current gold standard to diagnose active TB but have the disadvantage of being insensitive or time-consuming. The complicated pathogenesis of *M.tb* infection and limited knowledge on the mechanisms of host defense against this pathogen hinder the development of novel TB diagnostic tools and therapies.

The host immune system plays an important role in the development and control of disease during *M.tb* infection. Further study of the cellular and humoral immune responses involved will provide opportunities to investigate new immune strategies for diagnosis and therapy [7], [8]. One possible new area of research involves immunological biomarkers, for example, T-cell-based IFN-γ release assays (IGRAs) developed to identify *M.tb* sensitization [9], [10].

In addition to IFN-γ, there may be several cytokines and chemokines that have been investigated as potential biomarkers for *M.tb* infection and disease. For example, IL-4, IL-6, IL-10, CXCL10, CXCL8 and CCL8 levels are closely linked to active TB. It has also been suggested that distinct cytokine expression profiles in CD4^+^ T cells are associated with the bacterial loads characteristic of different infection states [11], [12]. Therefore, different multiplex immunological biomarker patterns may exist for either the different stages of latent tuberculosis infection (LTBI) or for different forms of tuberculosis. In this study, we attempted to identify characteristic profiles of cytokines or chemokines during *M.tb* infection and disease that may be important for new and more precise diagnostic methods.

Multi-cytokine/−chemokine measurements were performed using Luminex liquid array-based multiplexed immunoassays, which have been broadly applied for their sensitivity and ease of use. This technology has also been used in basic research to identify a number of disease biomarkers [13], [14], [15]. Therefore, we performed a comprehensive screen of 41 soluble immunological biomarkers, including 17 cytokines and 24 chemokines, to evaluate immunological biomarker profiles in the plasma or pleural effusions from patients with pulmonary TB and tuberculous pleurisy, as well as in plasma samples from latent tuberculosis infected persons. Our data demonstrated the presence of diverse biomarker profiles in latently infected individuals and TB patients. We also showed that the local immune environment of pleural effusions includes biomarkers that differ from those found in the peripheral blood. These results could be useful to better understand the immunopathology and immunoprotection of *M.tb* infection and disease. These data also indicated potential host immune biomarkers for novel diagnostic use.

## Materials and Methods

### Patients and Samples

All clinical samples were collected from Shenzhen Third People’s Hospital, China and Beijing Chest Hospital-Beijing Tuberculosis and Thoracic Tumor Research Institute, China. The diagnosis of pulmonary tuberculosis and tuberculous pleurisy was based on signs and symptoms, roentgenographic findings (chest X-ray and/or HRCT) consistent with TB and positive sputum bacterial examination ([16]). Clinical samples from untreated or less than one week treated pulmonary tuberculosis and tuberculous pleurisy patients were collected. Tuberculin skin test and a previously established *M. tuberculosis*–specific IFN-γ enzyme-linked immunospot (ELISPOT) [10] were performed on all recruited participants [17], [18]. Our study includes five clinical groups: (1) healthy donors (n = 12) without *M.tb* infection or contact history, (2) untreated pulmonary tuberculosis patients (n = 12), (3) latent tuberculosis infection individuals (n = 20), (4) tuberculous pleurisy patients (n = 15), and (5) lung adenocarcinoma patients (n = 15). The latent tuberculosis infection individuals were further separated into two groups based on the ELISPOT spot-forming cell (SFCs) counts of *M.tb* antigen stimulated wells: lower spot-forming cells (SFCs) counts (LTB1, n = 10, 30–80 SFCs) or higher SFCs counts (LTB2, n = 10, 110–400 SFCs). All subjects were over 18 years of age, sero-negative for HIV and HBV and did not have any autoimmune diseases (Table 1). HIV-infected individuals were excluded with an HIV antibody detection ELISA kit (WANTAI Biological Pharmacy Enterprise, China), and CD4^+^ T cell counts were analyzed on a flow cytometer using Expo32 software (Beckman Coulter, CA, USA). This work was approved by and conducted under the guidelines of the Ethical Committee of the Shenzhen–Hong Kong Institute of Infectious Disease, Shenzhen Third People’s Hospital. Written informed consent was obtained from all participants.

**Table 1:** Demographic Information.

Group		No.(n)	Sex (male/female)	Average Age	Clinical Diagnosis	ELISPOT SFCs
**HD**		12	7/5	30.7	Healthy	2–24
**Latent TB**	**LTB1**	10	4/6	40.7	Tuberculin skin test (TST) positive	34–97
	**LTB2**	10	5/5	46.1	Tuberculin skin test (TST) positive	110–400
**Active TB**	**TB**	12	5/7	38.5	Initially treated for pulmonary tuberculosis	110–400
	**TP**	15	9/6	32.8	Tuberculous pleurisy	12–476
**Lung cancer**		15	6/9	54.3	Adenocarcinoma (AC)	–

For the plasma collection, EDTA was used as an anticoagulant. After collecting blood, the plasma was removed by centrifugation, and PBMCs were isolated by Ficoll density gradient centrifugation. PFMCs were obtained from the pleural fluid by centrifugation at 300 g for 5 min. The viability of both PBMCs and PFMCs was analyzed by Trypan Blue staining as described in publication [19]. The cell viability was greater than 90% in each sample.

### Multi-cytokine/Chemokine Measurements

In total, 41 soluble immunological molecules were tested (Table 2). A panel of 17 cytokines was measured using the Bio-Plex Pro Cytokine Assay, 17-Plex Group I kit (Bio-Rad Laboratories, USA). Chemokine detection was performed using a Human Cytokine/Chemokine Panel (MPXHCYTO-60K, MPXHCYP2-62K and MPXHCYP3-63K, Millipore, USA). Assays were performed according to the manufacturers’ instructions. Data were collected and analyzed using Luminex xPONENT software (Luminex Corporation, USA). A five-parameter regression formula was used to calculate the sample concentration from the standard curves. The Human Cytokine Quality Controls 1 and 2 included in the kit were used as low and high concentration quality controls.

**Table 2:** Classification of examined cytokines and chemokines.

**Chemokine**	CCL	CCL1/I-309, CCL2/MCP-1, CCL3/MIP-1α, CCL4/MIP-1β, CCL5/RANTES, CCL7/MCP-3, CCL8/MCP-2, CCL11/Eotaxin, CCL13/MCP-4, CCL15/MIP-1δ, CCL17/TARC, CCL20/MIP-3α, CCL21/6Ckine, CCL24/Eotaxin-2, CCL26/Eotaxin-3, CCL27/CTACK
	CXCL	CXCL5/ENA-78, CXCL6/GCP-2/LIX, IL-8/CXCL8, CXCL9/MIG, IP-10/CXCL10, CXCL11/I-TAC, CXCL12/SDF-1α+β, CXCL13/BCA-1
**TNF**		TNFα
	Type I	IL-2, IL-15, IL-4, IL-13, IL-7, IL-9, IL-5, GM-CSF, IL-6, IL-12, G-CSF
**Interleukin**	Type II	IL-10, IFN-γ
	Ig superfamily	IL-1RA, IL-1β
	IL-17 family	IL-17

### PBMCs and PFMCs Stimulation in vitro

For *in vitro* cell stimulation, either antigens were extracted from the bacterial strain H37Rv (Prof. Z D Zhang kindly provided) or purified ESAT-6 protein (kindly provided by Professor X CH Chen) was used. The H37Rv bacteria were inactivated by heating to 65°C for 20 minutes, and the bacterial cells were lysed by sonication in an ice-water bath. The total protein concentration of the H37Rv lysate was quantified by a Bradford assay.

PBMCs from all participates were collected by Ficoll centrifugation using a published protocol [20], [21]. PFMCs from tuberculous pleurisy and cancer patients were collected by centrifugation. The cells were plated at a density of 5×10^5^ cells/well in 96-well plates with or without H37Rv lysate or ESAT-6 antigen at a final concentration of 10 µg/ml. Un-stimulated cells and cells treated with an anti-CD3/CD28 polyclonal cocktail (1 µg/ml) were used as negative and positive controls, respectively. After 72 hours incubation in a 37°C, 5% CO_2_ incubator, the culture supernatants were collected by centrifugation and used for multi-cytokine/chemokine measurements. [22], [23], [24], [25].

### Data Analysis

GraphPad Prism 5 (GraphPad software) was used to generate plots and perform statistical analyses on the data. The statistical analysis was completed using SPSS. First, the data were analyzed by a one-way analysis of variance (ANOVA). When the variance between samples was equal, a Tukey’s HSD or LSD post-hoc test was performed. When the variance was not equal, the Tambane or Dunnett T3 method was used to determine whether there were significant differences between two groups. All the data are presented as the mean ± SEM, and a difference with p<0.05 was considered to be significant. The correlation coefficient was assessed using Pearson’s two-tailed correlation test.

## Results

### 1. Individuals with Latent Tuberculosis Infection and a Pattern of Enhanced IFN-γ Secretion Showed Significant IL-2, IP-10, CXCL11 and CXCL12 Expression

An *M. tuberculosis*–specific IFN-γ enzyme-linked immunospot (ELISPOT) assay is helpful to differentiate between individuals with LTBI and truly healthy individuals but cannot distinguish between latent infection and active TB [10], [17], [18]. We found that LTBI individuals had variable ELISPOT SFC values, and some of them even displayed an *M.tb* antigen-specific ELISPOT SFC counts as high as those observed in TB patients. As a result, a more comprehensive analysis is needed to screen for new biomarkers.

**Figure 1:**
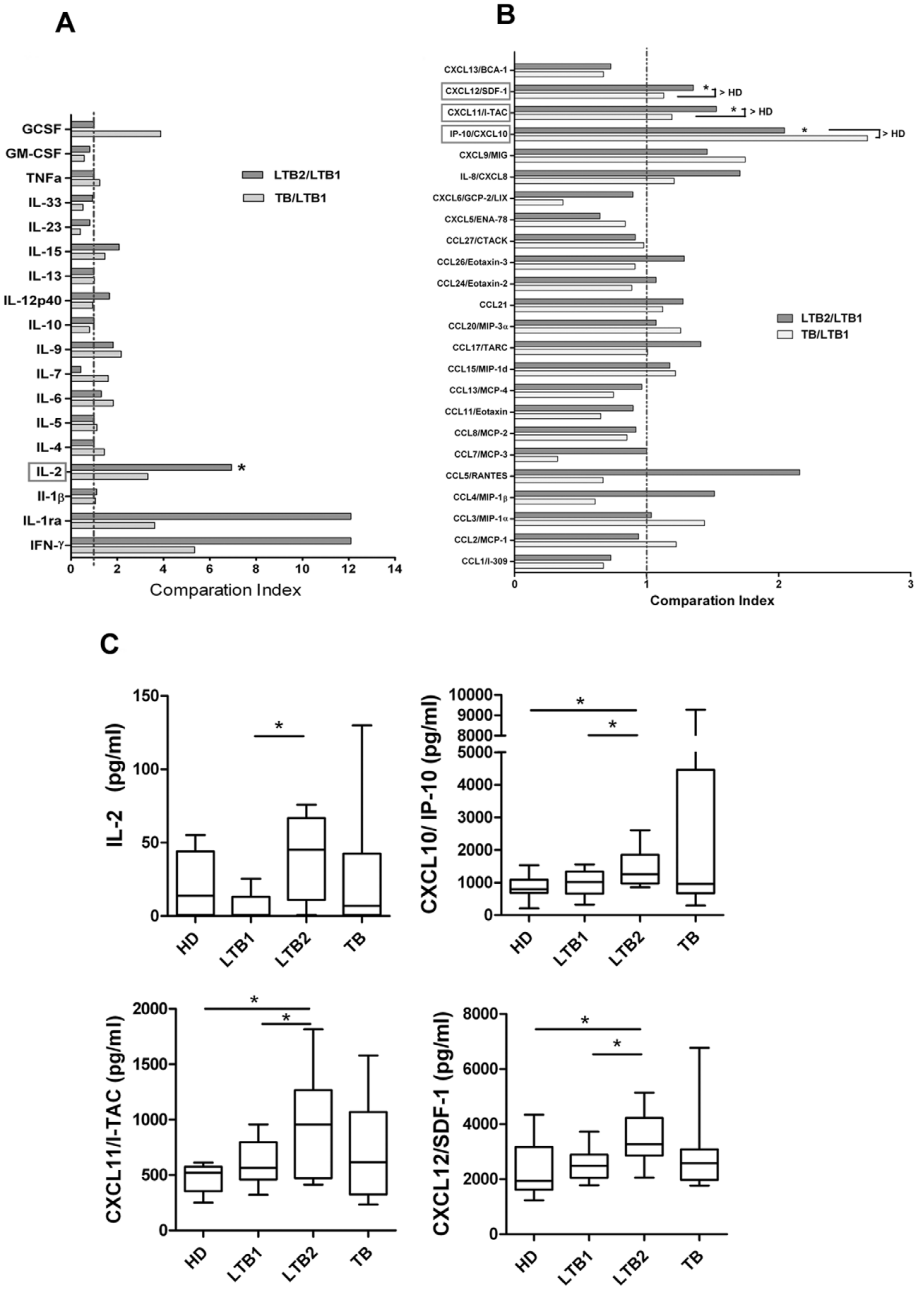
Characterization of cytokine/chemokine expression in LTBI high IFN-γ or low IFN-γ groups. A and B: The Comparison Index was calculated for each cytokine and chemokine by dividing the concentration of the cytokine/chemokine in the LTBI high IFN-γ (LTB2) or active pulmonary tuberculosis (TB) group by the concentration of the cytokine/chemokine in the LTBI low IFN-γ group (LTB1). LTBI individuals were separated into two groups based on *M.tb*-specific ELISPOT spot-forming cells counts: lower SFCs counts (LTB1, n = 10, 30–80 SFCs) and higher SFCs counts (LTB2, n = 10, 110–400 SFCs) by *M.tb* antigen-specific IFN-γ ELISPOT assay (ESAT-6 protein or ESAT-6/CFP-10-derived peptide pools as stimulants). A value greater than 1 indicates that the concentration of the cytokine/chemokine in the LTB2 or TB group was higher than that of LTB1 group. An asterisk indicates that a significant difference was observed between the two groups. “>HD” means both the LTB1 and TB were higher than healthy donor group (HD). **C:** Expression levels of 4 molecules (IL-2, IP-10, CXCL11 and CXCL12) that differ significantly between the LTB1 and LTB2 groups are shown. Horizontal bars represent median values, boxes represent the interquartile range (25–75%) and whiskers represent the highest and the lowest values. Horizontal lines indicate a statistically significant difference between groups. *p<0.05.

In our study, LTBI donors were separated into two groups as suggested by apparent differences observed using the IFN-γ ELISPOT test. One cluster of donors had low-level responses by ELISPOT assay, yielding SFCs counts between 30 and 80 (designated LTB1). In contrast, the other cluster of donors yielded SFCs counts between 110 and 400 (designated LTB2). Not all of the molecules tested were equivalently expressed in these two groups of donors. The cytokine interleukin-2 and the chemokines IP-10, CXCL11 and CXCL12 were significantly higher in the LTB2 group, as demonstrated by their high ELISPOT results. A Comparison Index was used to demonstrate the significant differences (Figure 1, A and B). The Comparison Index is defined as the ratio of the concentration of a cytokine/chemokine in the LTB2 (or TB) group to that observed in the LTB1 group. As such, a ratio above 1 signifies that the concentration of the cytokine/chemokine in the LTB2 (or TB) group was higher than the concentration of the cytokine/chemokine in the LTB1 group. The differences between LTB2 and LTB1 were only significant for IL-2, IP-10, CXCL11 and CXCL12. Moreover, the levels of IP-10, CXCL11 and CXCL12 expression in LTB2 plasma were not significantly different from the levels found in pulmonary tuberculosis patients but were much higher than the levels detected in the healthy control group (Figure 1C). These data may suggest that an individual with latent tuberculosis infection who has a high value on the ELISPOT assay may exhibit a similar immune response to an active pulmonary tuberculosis patient, this phenomena is consistent with our previous microarray analysis on CD4^+^ T cells (data have been published in reference [26]).

### 2. Overlapping Cytokine/Chemokine Expression Patterns were Observed in Patients with Active Tuberculosis and Tuberculous Pleurisy

**Figure 2:**
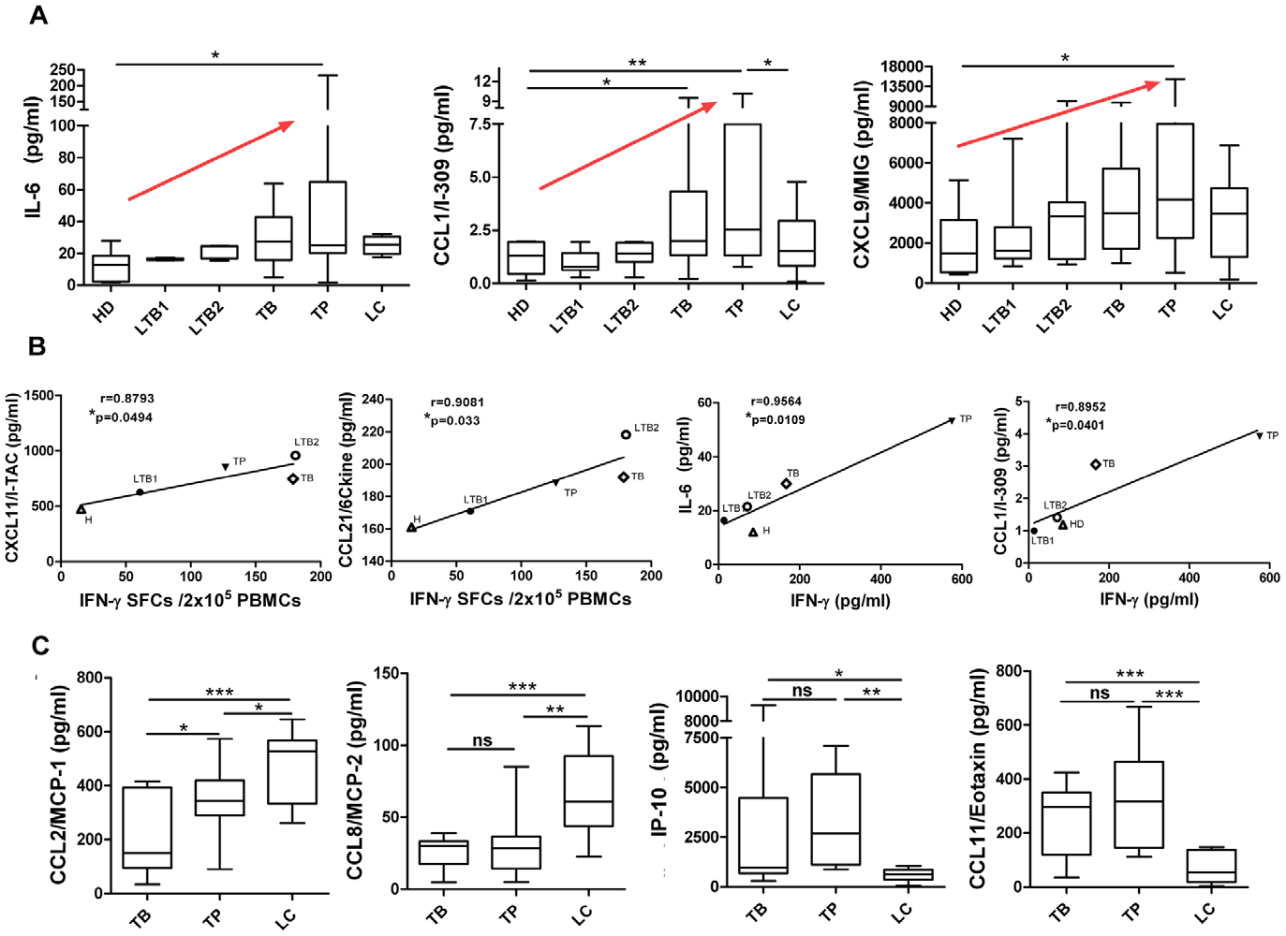
Differences in cytokine/chemokine expression among distinct clinical groups. **A:** IL-6, CCL1 and CXCL9 levels in plasma from healthy donors, LTB1 and LTB2 individuals, active TB patients, tuberculous pleurisy patients and lung cancer patients. A red arrow indicates an upward trend. **B:** Correlation analysis between CXCL11 and CCL21 concentrations in the HD, LTB1, LTB2, TB and TP groups and the IFN-γ ELISPOT SFC values; and the correlation analysis between IL-6 and CCL1 concentrations and IFN-γ plasma concentration. The correlation coefficients were assessed using Pearson’s two-tailed correlation test. **C:** CCL2, CCL8, IP-10 and CCL11 levels in active pulmonary TB, tuberculous pleurisy and lung cancer patients. Horizontal bars represent median values, boxes represent the interquartile range (25–75%) and whiskers represent the highest and the lowest values. Horizontal lines indicate a statistically significant difference between groups. *p<0.05; **p<0.005.

Figure 2A shows that the expression of IL-6, CCL1 and CXCL9 in plasma was increased in active pulmonary tuberculosis and tuberculous pleurisy patients compared with healthy controls. Because elevated IFN-γ is considered to be a marker of *M.tb* infection, we further analyzed the correlation coefficient of different groups with IFN-γ SFCs or IFN-γ plasma concentration, as shown in Figure 2B. A significant positive linear correlation with IFN-γ SFCs was observed for CXCL11 and CCL21, whereas a significant correlation with IFN-γ plasma concentration was found for IL-6 and CCL1.

Lung cancer patients were used to model a different type of lung disease as a control. Patients with tuberculous pleurisy were also analyzed separately from the pulmonary tuberculosis patients. We found that CCL8, CCL11 and IP-10 did not significantly differ between pulmonary tuberculosis and tuberculous pleurisy patients. However, these three chemokines were significantly different in these TB groups compared with the lung cancer control group. Specifically, the plasma CCL8 levels in lung cancer patients were much higher than the levels observed in either pulmonary tuberculosis patients or tuberculous pleurisy (TP) patients. In contrast, CCL11 and IP-10 levels in lung cancer patients were much lower than the levels detected in TB and TP groups (Figure 2C). Figure 2C illustrates a similar expression pattern for CCL2 and CCL8, except that a significant difference between TB and TP patients was also noted. Based on these results, we may surmise that CCL11 and IP-10 are specifically increased in plasma from pulmonary tuberculosis and tuberculous pleurisy patients, while CCL8 and CCL2 are specifically decreased when compared with lung cancer controls. These results strongly indicate a specific chemokine pattern in the plasma of pulmonary tuberculosis patients and tuberculous pleurisy that is distinct from the pattern observed in lung cancer patients.

### 3. Elevated IL-6 and IL-9 in the Periphery of Active Pulmonary Tuberculosis Patients were Antigen Specific

**Figure 3:**
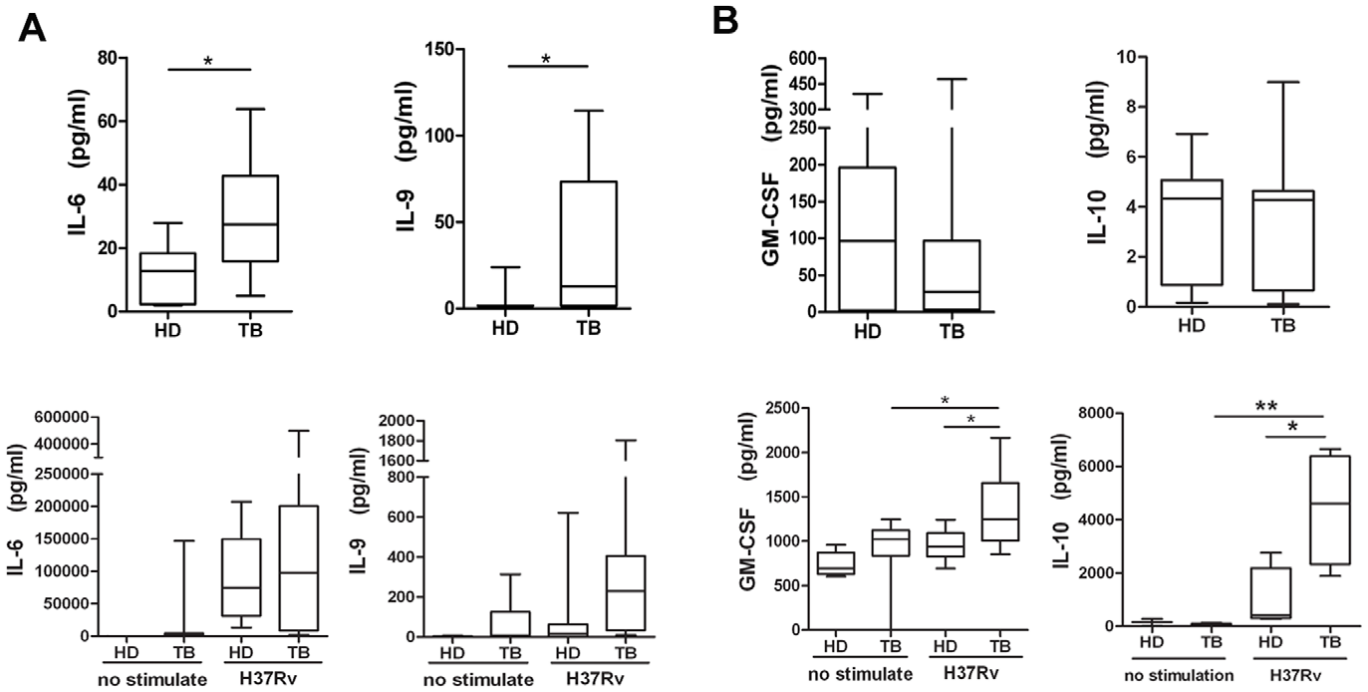
Comparison of cytokine expression in plasma or *in vitro* after *M.tb* antigen-specific stimulation of PBMCs. **A:** Data for IL-6 and IL-9 levels in pulmonary tuberculosis patients (TB) and HDs are shown. PBMCs from healthy donors and TB patients were collected for *M.tb* antigen-specific stimulation *in vitro*. The IL-6 and IL-9 expression levels in stimulated cell cultures are shown in the panels below. **B:** GM-CSF and IL-10 levels in plasma from TB patients and healthy donors or in PBMCs following antigen-specific stimulated cell culture. Horizontal bars represent median values, boxes represent the interquartile range (25–75%) and whiskers represent the highest and the lowest values. Horizontal lines indicate a statistically significant difference between groups. *p<0.05; **p<0.005.

Compared with healthy donors, the peripheral blood plasma of pulmonary tuberculosis patients contained significantly higher levels of IL-6 and IL-9 (Figure 3A). To evaluate the possible causes of increased cytokine levels, peripheral blood mononuclear cells (PBMCs) from either HDs or pulmonary tuberculosis patients were collected and stimulated *in vitro* with the H37Rv-derived *Mycobacterium tuberculosis* antigen. Un-stimulated and polyclonal anti-CD3/CD28-stimulated cells were used as negative and positive controls, respectively. The ability of *M.tb* antigens to increase the production of these cytokines was assessed. We found that either specific antigen could stimulate PBMCs from pulmonary tuberculosis patients to produce higher levels of IL-6 and IL-9 than PBMCs from HDs. These data suggest that high levels of IL-6 and IL-9 may be released following stimulation by *M.tb*. Finally, we determined that GM-CSF and IL-10 levels increased markedly after antigen stimulation *in vitro* although no differences were detected in plasma samples (Figure 3B). Higher expression levels of several chemokines, including CCL-8, CXCL13, CXCL12, CCL1 and CCL21, were only induced in PBMCs after stimulation with an *M.tb*-specific antigen (either H37Rv lysate or purified ESAT-6 protein) (Figure S3). Our data also showed a consistent antigen-related secretion pattern following either H37Rv lysate or purified ESAT-6 protein stimulation.

### 4. Pleural Effusions from Patients with Tuberculous Pleurisy Exhibited a Different Antigen-specific Cytokine/Chemokine Secretion Pattern Compared with those from Lung Cancer Patients

**Figure 4:**
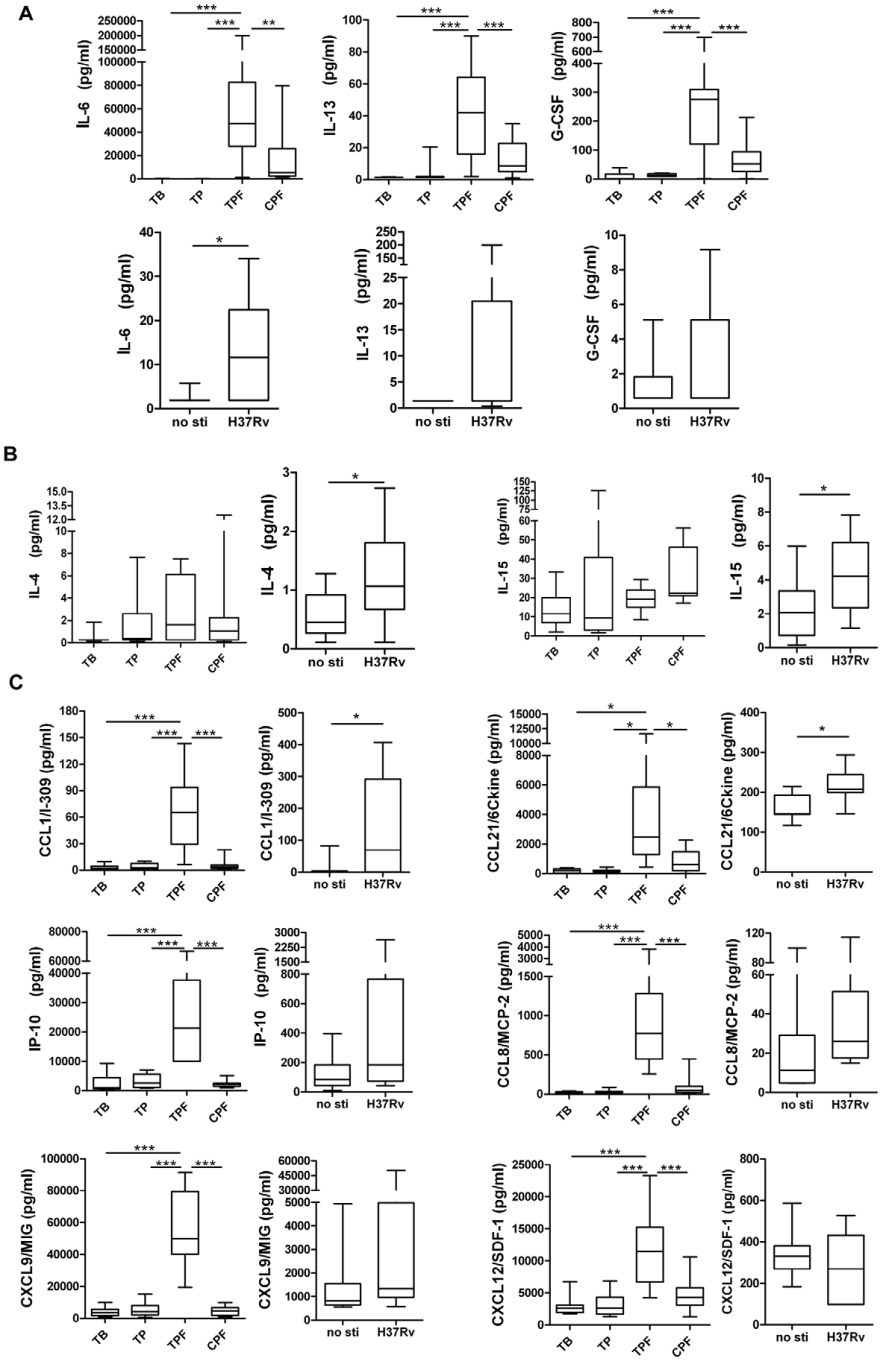
Comparison of cytokine/chemokine expression in plasma or pleural effusions or *in vitro* after *M.tb* antigen-specific stimulation of PFMCs. **A.** Levels of IL-6, IL-13 and G-CSF in plasma from active pulmonary tuberculosis (TB) and tuberculous pleurisy (TP) patients or the pleural effusions from tuberculous pleurisy (TPF) and lung cancer (CFP) patients. PFMCs from tuberculous pleurisy patients were collected for *M.tb* antigen-specific stimulation *in vitro*. The levels of IL-6, IL-13 and G-CSF in stimulated cell cultures are shown in the panels below. **B:** IL-4 and IL-15 levels in plasma or pleural effusions (above) and in PFMCs antigen-specific stimulated cell cultures (below). **C:** Chemokine levels in the plasma or pleural effusions (lines 1 and 3) and in antigen-specific stimulated PFMCs cultures (lines 2 and 4). Horizontal bars represent median values, boxes represent the interquartile range (25–75%) and whiskers represent the highest and the lowest values. Horizontal lines indicate a statistically significant difference between groups. *p<0.05; **p<0.005; *** p<0.0005.

Tuberculous pleurisy usually develops soon after an initial infection. In tuberculous pleurisy, the amount of fluid in the pleural space increases dramatically following the bacterial invasion of the space. The pleural effusions contain cells, primarily lymphocytes, and extracellular proteins, including cytokines and chemokines, which may reflect a local immune response. Our data shown that G-CSF, IL-6 and IL-13 levels were significantly higher in tuberculous pleurisy pleural effusions (TPF) compared with plasma from TB or TP patients. Furthermore, the expression of these cytokines was distinctly increased compared with the pleural effusions from lung cancer patients (CPF) (Figure 4A), suggesting that enhanced expression of these three cytokines could be tuberculosis specific. Using *M.tb* antigen to stimulate PFMCs from TP patients *in vitro* resulted in increased expression of cytokines, particularly IL-6, which showed a significant change after stimulation (Figure 4A). These data may suggest that infiltrating lymphocytes were the source of increased cytokine production in the pleural effusions.

IL-4 and IL-15 expression levels were increased *in vitro* by the antigenic stimulation of PFMCs; they also exhibited a unique expression pattern in the pleural effusions of TP patients, as shown in Figure 4B. Therefore, the PFMCs from tuberculous pleurisy patients have the ability to produce IL-4 and IL-15 when stimulated with *M.tb*.

To further understand the local inflammatory situation, expression of chemokines was detected. CCL1, CCL21, IP-10, CCL8 and CXCL9 levels were much higher in pleural effusions from patients with TP compared with lung cancer patients. Similarly, stimulation of PFMCs with antigen increased their expression of chemokines *in vitro* (Figure 4C). CXCL12 was highly expressed in the TP pleural effusions but not after stimulation, which may result from the kinetics of cytokine production or from a separate production pathway. (Figure 4C).

### 5. The TB Disease-related Cytokine/Chemokine Secretion Pattern was Correlated with Antigen-specific IFN-γ Secretion

**Figure 5:**
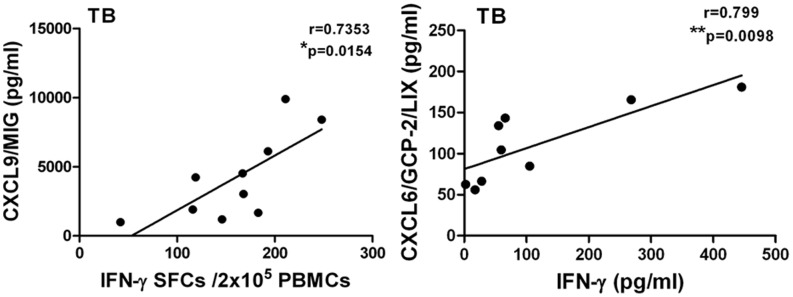
Correlation analysis between CXCL9 and CXCL6 concentrations and IFN-γ ELISPOT SFC values or IFN-γ plasma concentration. The correlation coefficient was assessed using Pearson’s two-tailed correlation test.

IFN-γ is the only soluble immunological marker currently used for helping identify *M.tb* infection. However, IFN-γ is not useful to distinguish latent from active disease. Other cytokines and chemokines whose expressions are correlated with IFN-γ may be useful to characterize *M.tb* infection and disease. Therefore, we further analyzed the correlation between the concentrations of cytokines and chemokines in plasma from active pulmonary tuberculosis or tuberculous pleurisy patients and the number of IFN-γ spot-forming cells in an ELISPOT assay or the IFN-γ concentration in plasma. These results showed that the expression of IL-1RA, IL-5, IL-7 and IL-13 in plasma from both pulmonary tuberculosis and tuberculous pleurisy patients significantly correlated with their IFN-γ expression (Table 3, 4). Figure 5 shows that significant correlations were found only in the TB group, for whom CXCL9 was correlated with IFN-γ SFCs and CXCL6 was correlated with IFN-γ plasma concentration.

**Table 3:** Cytokines showing significant correlations with IFN-γ in TB plasma.

TB	IL-1RA	IL-5	IL-13	IL-7	IL-12 (P70)	IL-9	IL-2
**Pearson r**	0.9004	0.7560	0.7915	0.7550	0.6918	0.7625	0.8254
**P value (2-tailed)**	0.0002	0.0071	0.0037	0.0072	0.0183	0.0064	0.0018
**sig.**	**	**	**	**	*	**	**

**Table 4:** Cytokines showing significant correlations with IFN-γ in tuberculous pleurisy plasma.

TP	IL-1RA	IL-5	IL-13	IL-7	IL-4	IL-10	IL-15	TNF-α	IL-1β
**Pearson r**	0.6188	0.9470	0.9446	0.9465	0.9303	0.7636	0.5995	0.9531	0.9334
**P value (2-tailed)**	0.0241	<0.0001	<0.0001	<0.0001	<0.0001	0.0024	0.0303	<0.0001	<0.0001
**sig.**	*	***	***	***	***	**	*	***	***

*** .Correlation is significant at the 0.0001 level (2-tailed).

** .Correlation is significant at the 0.01 level (2-tailed).

* .Correlation is significant at the 0.05 level (2-tailed).

## Discussion

Few reports have been published that examine the detection of a large number of immune biomarkers and compare them across TB-related diseases and latency. We performed a comprehensive screen of 41 soluble immunological biomarkers, including 17 cytokines and 24 chemokines in samples from different groups. The demographic data for the patients included in this study are shown in a supporting table (Table S1). Our results showed that potentially informative immune markers have different expression in patients with latent tuberculosis infection, active pulmonary TB and tuberculous pleurisy. Although T-cell based IFN-γ release assays (IGRAs) were developed to assess IFN-γ production and to demonstrate sensitization to *M.tb* antigens after *in vitro* stimulation with *M.tb*-specific immunodominant antigens, IGRAs cannot distinguish active TB from latency [9]. A majority of patients with a positive IGRA will not progress to active TB, and these individuals may not need preventive therapy [27]. According to published reports [28], [29], [30], Pai et al believed that IGRA testing results exhibit dynamic variability [27], revealing underlying phenotypic differences that may predict different disease outcomes. Therefore, a single IGRA test may never predict the infection progression; rather, it is possible for the IGRA test, in combination with other complementary markers, to provide a more accurate diagnosis or prediction of disease. We analyzed the correlation of multiple soluble immune molecules with *M.tb* antigen specific IFN-γ SFCs by ELISPOT or IFN-γ concentrations in plasma. Cytokine/chemokine assays showing significant correlation with IFN-γ may identify a potential biomarker for use in combination with IFN-γ to distinguish latency and active disease clinically (shown in Figure 5; LTBI-related data not shown).

In our study, an analysis of two groups of latent TB-infected subjects (identified based on *M.tb* antigen specific IGRA result) was performed to determine whether they also differed in their plasma cytokine expression levels. For example, this analysis showed that individuals with a high frequency of IFN-γ-producing cells had significantly higher expression levels of IL-2, IP-10, CXCL11 and CXCL12 in their plasma compared with healthy controls. The latent group with a lower frequency of IFN-γ producing cells displayed a pattern similar to that of the active TB patient group. Approximately one-third of the world’s population is infected with *M.tb*, although only approximately 10% ever develop active tuberculosis. In contrast, the remaining 90% of infected individuals maintain a latent state [1], [2]. This large group of people is at an increased risk of developing active tuberculosis and becoming infectious. As a result, it is important to identify and monitor this group of individuals and perform intervention therapy promptly. Some researchers have focused on identifying biomarkers for distinguishing LTBI from active TB, generally using gene expression profiles [31], [32]. However, other studies distinguish latency based on other immune criteria. For example, it is estimated that 15% of the Chinese population is latently infected, as identified by T-cell-based gamma interferon release assays (IGRAs) [33]. However, clinical diagnosis that relies on IFN-γ ELISPOT test may be limited. Our results indicated that LTBI patients had different plasma cytokine and chemokine levels based on a correlation analysis with their *M.tb* antigen-specific IFN-γ producing ability by ELISPOT assay. Therefore, these distinct soluble immunological factors correlate with *M.tb* antigen-specific IFN-γ release assay might be useful to help distinguish latent tuberculosis infection and tuberculosis disease.

Although host immune factors play a pivotal role in the control of *M.tb* infection, many immunological signatures of disease progress are still unknown [34], [35], [36], [37]. Johannes Nemeth et al. have reported the specific cytokine patterns of pulmonary tuberculosis in central Africa. They detected a pronounced pro-inflammatory cytokine response in patients, with highly significantly increased levels of IL-6 and TNF-α accompanied by increased TGF-β [38]. Similarly, we found a significant increase in the plasma levels of IL-6 and IL-9 in patients with active pulmonary tuberculosis and tuberculous pleurisy. In addition, IL-6 was specifically increased in pleural effusions from patients with tuberculous pleurisy compared with the pleural effusions of lung cancer patients. IL-6 has been reported to participate in the immunopathogenesis of tuberculosis. IL-6 is required for an initial protective IFN-γ response during early *M.tb* infection. This proinflammatory cytokine is mainly produced by monocytes after various bacterial infections [39]. T. C. Y. Tsao et al. found significantly higher levels of IL-6 in the bronchoalveolar lavage fluid (BALF) from patients with active pulmonary tuberculosis and IL-6 release by alveolar macrophages from TB lesions [40]. Our results also provide evidence for the relationship between IL-6 and untreated active TB, where IL-6 may be produced by monocytes in the peripheral blood or at the local site. It has also been reported that this early response occurs in the lungs and is important for the initial restriction of mycobacterial growth [39]. However, the precise mechanism by which IL-6 mediates protection needs to be further clarified. Increased expression of IL-9 may contribute to the development of TB, as it is associated with an impaired Th1 immune response in patients with tuberculosis [41], [42].

In addition, our results suggested a new tool that could be used in a tuberculous pleurisy diagnostic test. There are several diagnostic methods used in clinics, such as the acid-fast bacilli test of pleural fluid or histological analysis and mycobacterial culture of closed pleural biopsied tissue [43], [44]. In addition to these standard methods, other pleural fluid biomarkers have been investigated for their diagnostic potential, including adenosine deaminase (ADA), IFN-γ and soluble Fas ligand (sFasL) [45], [46]. Herein, we found several unreported cytokines and chemokines were expressed at significantly high levels in tuberculous pleurisy patients. The most noteworthy are a group of soluble molecules that are highly expressed specifically in pleural effusions from tuberculous pleurisy patients but not lung cancer patients. Additionally, expression of these soluble molecules is antigen specific. Our data show that CCL1, CXCL9 and IP-10 were highly expressed in both the periphery and the pleural effusions from tuberculous pleurisy patients. Moreover, among the examined factors, CCL1, CCL21 and IL-6 were markedly increased in pleural effusions from tuberculous pleurisy patients and the supernatants of cultured PFMCs after *M.tb*-specific antigen stimulation. These findings suggest that these soluble molecules may be useful as a panel of immune biomarkers for the diagnosis of tuberculous pleurisy and helping proper treatment. An intensive study on mechanism underlying antigen-specific immunological factors secretion and immune pathogenesis and protection still needs further understanding, which will undoubtedly improve clinical management.

Chemokines belong to a large family of proteins called chemotactic cytokines and have an average molecular mass of 8–14 kDa. They can mediate the constitutive recruitment of leukocytes from the blood into tissues [47]. Chemokines are generally separated into two families: the CC family functions to attract and activate monocytes/macrophages, lymphocytes, basophils, eosinophils, NK cells and dendritic cells, whereas CXC chemokines mainly attract and activate neutrophils and some activated T cells or NK cells [48]. In this study, we assayed a broad range of CC and CXC chemokines and found that some of them were significantly expressed during *M.tb* infection, including CCL1 and CXCL9, which have not been previously studied. Zahra Hasan et al. showed that the relationship between mycobacterial antigen-induced IFN-γ and CXCL9 may play a role in determining disease severity in tuberculosis [49]. However, the effects of increased CCL1 production during *M.tb* infection are not yet reported.

Some chemokines have been investigated in the context of *M.tb* infection and have been shown to participate in protective and immunopathologic host responses during human tuberculosis [50]. In general, the production of chemokines is essential for the recruitment of inflammatory cells at the site of infection and the formation and maintenance of a granuloma. It has been reported that RANTES and MCP-1 are elevated in the bronchoalveolar lavage fluid of tuberculosis patients [51]. MCP-1 is considered to be a potent immunoprophylactic tool for controlling the mycobacterial colonization of the host [52]. Our data also show that MCP-1 was highly expressed in pleural effusions from patients with tuberculous pleurisy, although its expression level did not differ from a carcinomatous pleural effusions control (Figure S1), suggesting that this maybe not be a unique response to TB. In addition, the level of RANTES in tuberculous pleurisy pleural effusions in our research was decreased significantly compared with that in control plasma (Figure S2). These differences may reflect the unique characteristics of samples from different sources, i.e., pleural effusions rather than bronchoalveolar lavage fluid with no healthy donor comparisons. Furthermore, the CC chemokines IP-10 and MCP-2 are potentially ideal candidate biomarkers because of their inducible *in vitro* antigen-specific expression, and IP-10 is a potential diagnostic marker for evaluating tuberculous pleurisy [53], [54]. CCL21 was reportedly induced in the lungs and secreted within granulomatous lesions after infection with *M. tuberculosis* [55], and our finding that CCL21 expression was enhanced in tuberculous pleurisy pleural effusions is consistent with this conclusion. For CXCL8, another important CXC chemokine in TB, we also found that it was expressed at high levels in both tuberculous pleurisy pleural effusions and carcinomatous pleural effusions (Figure S1). It has been reported that CXCL8 might be involved in eliciting the initial immune response to mycobacterial antigens by homing and infiltrating Th1 cells and that pleural macrophages might be the major source of this chemokine [56]. CXCL12 levels are also increased specifically in tuberculous pleurisy pleural effusions, although the mechanism and function of this increase have not been demonstrated.

In summary, we characterized varied and antigen-specific cytokine and chemokine patterns in latent tuberculosis infection, active pulmonary tuberculosis and tuberculous pleurisy patients. These results strongly implicate the potential for a combination of different immunological factors with IFN-γ to distinguish between latent TB, active pulmonary tuberculosis and tuberculous pleurisy, which may lead to a more accurate diagnosis. Furthermore, panel of immunological factor candidates need to be selected and further evaluated in a larger number of patients in the following study. Those researches will be useful for a better clinical practice and a better understanding of their expression and regulation during *M.tb* infection and disease.

## Supporting Information

Figure S1 **Elevated cytokines/chemokines in pleural effusions from both tuberculous pleurisy and lung cancer patients.** Comparison of IL-10, CXCL13, CCL2 and CXCL8 expression in plasma from active pulmonary tuberculosis and tuberculous pleurisy patients and pleural effusions from tuberculous pleurisy and lung cancer patients. Expression levels were increased in both tuberculous pleurisy pleural effusions and pleural effusions from lung cancer patients. Horizontal bars represent median values, boxes represent the interquartile range (25–75%) and whiskers represent the highest and the lowest values. Horizontal lines indicate a statistically significant difference between groups. *p<0.05; **p<0.005; *** p<0.0005.
(TIF)

Figure S2 **Decreased cytokines/chemokines in pleural effusions.** IL-2, CXCL5, CCL5 and CCL27 expression in plasma from active pulmonary tuberculosis and tuberculous pleurisy patients or pleural effusions from tuberculous pleurisy and lung cancer patients. These data revealed decreased expression levels in tuberculous pleurisy pleural effusions compared with plasma. Horizontal bars represent median values, boxes represent the interquartile range (25–75%) and whiskers represent the highest and the lowest values. Horizontal lines indicate a statistically significant difference between groups. *p<0.05; **p<0.005; *** p<0.0005.
(TIF)

Figure S3 **Antigen-specific responses in PBMCs.** IL-10, CCL-8, CXCL13, CXCL12, CCL1 and CCL21 were highly expressed by PBMCs from active pulmonary tuberculosis patients after stimulation with either H37Rv lysate or ESAT-6 protein. The data show that both H37Rv lysate and ESAT-6 protein stimulated PBMCs to release antigen-specific cytokines/chemokines. Horizontal bars represent median values, boxes represent the interquartile range (25–75%) and whiskers represent the highest and the lowest values. Horizontal lines indicate a statistically significant difference between groups. *p<0.05; **p<0.005; *** p<0.0005.
(TIF)

Table S1 **Cytokine/chemokine concentrations in different groups.**
(DOC)
