# Supplementary figures and images for: Different Patterns of Cytokines and Chemokines Combined with IFN-γ Production Reflect Mycobacterium tuberculosis Infection and Disease
Source: PLoS One. 2012 Sep 13;7(9):e44944. doi: 10.1371/journal.pone.0044944 (PMC3441719; doi:10.1371/journal.pone.0044944)

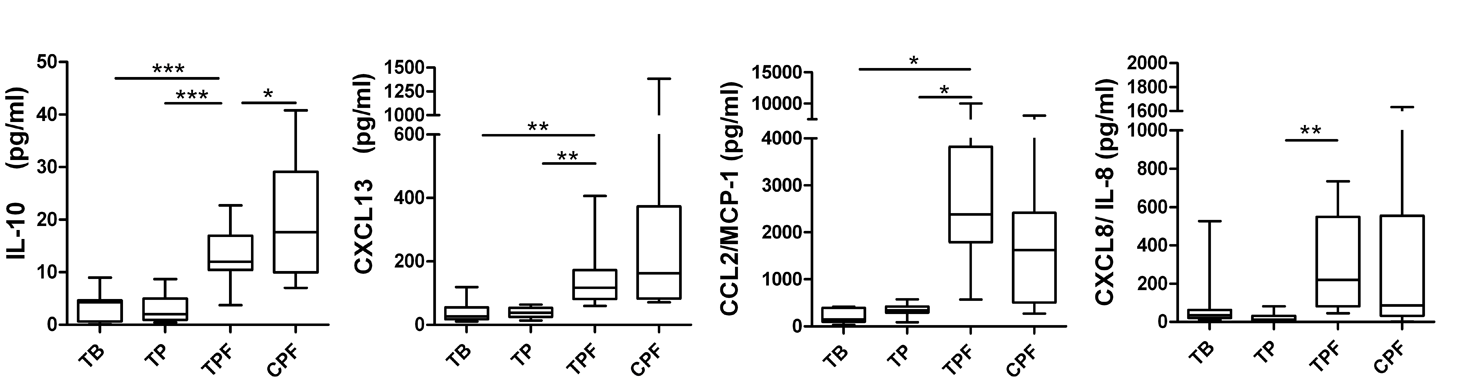

Supplement: Figure S1 — Elevated cytokines/chemokines in pleural effusions from both tuberculous pleurisy and lung cancer patients. Comparison of IL-10, CXCL13, CCL2 and CXCL8 expression in plasma from active pulmonary tuberculosis and tuberculous pleurisy patients and pleural effusions from tuberculous pleurisy and lung cancer patients. Expression levels were increased in both tuberculous pleurisy pleural effusions and pleural effusions from lung cancer patients. Horizontal bars represent median values, boxes represent the interquartile range (25–75%) and whiskers represent the highest and the lowest values. Horizontal lines indicate a statistically significant difference between groups. *p<0.05; **p<0.005; *** p<0.0005. (TIF) [file pone.0044944.s001.tif]

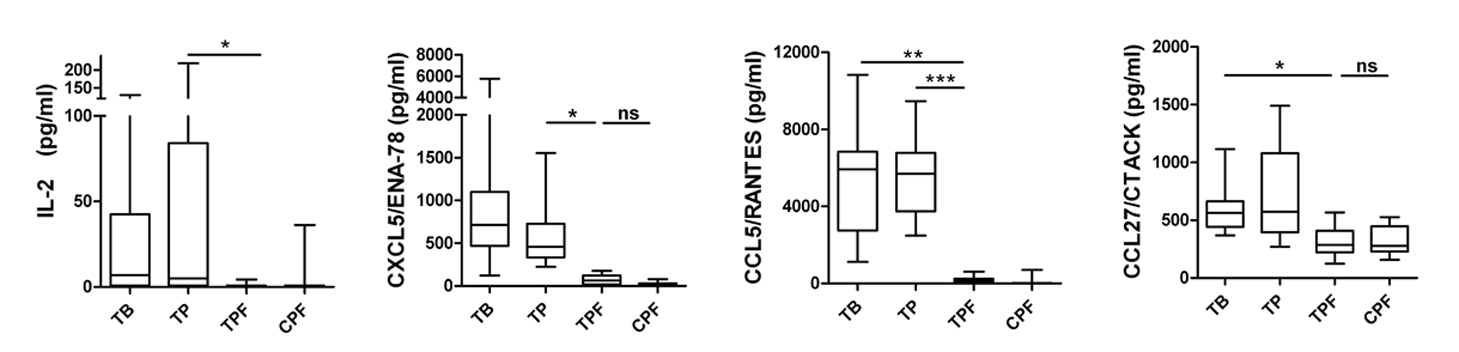

Supplement: Figure S2 — Decreased cytokines/chemokines in pleural effusions. IL-2, CXCL5, CCL5 and CCL27 expression in plasma from active pulmonary tuberculosis and tuberculous pleurisy patients or pleural effusions from tuberculous pleurisy and lung cancer patients. These data revealed decreased expression levels in tuberculous pleurisy pleural effusions compared with plasma. Horizontal bars represent median values, boxes represent the interquartile range (25–75%) and whiskers represent the highest and the lowest values. Horizontal lines indicate a statistically significant difference between groups. *p<0.05; **p<0.005; *** p<0.0005. (TIF) [file pone.0044944.s002.tif]

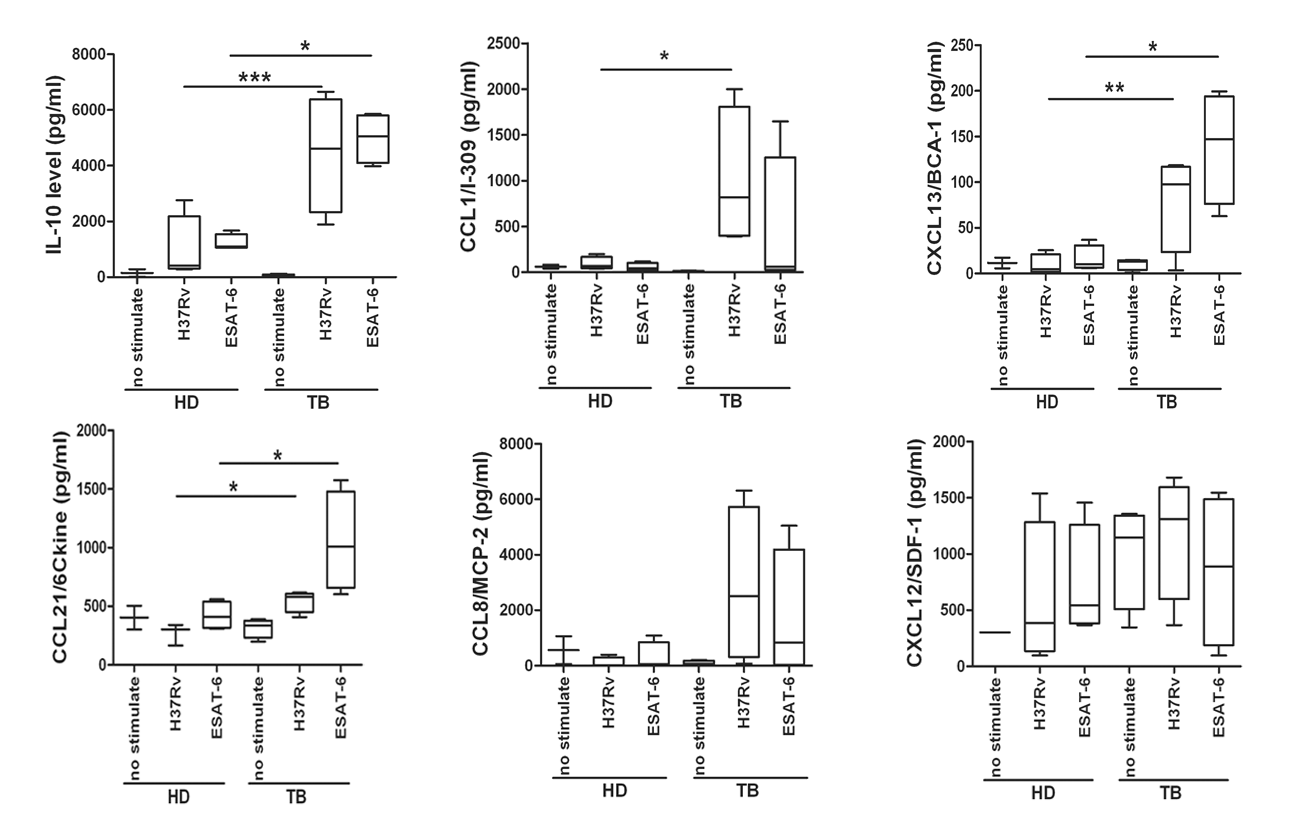

Supplement: Figure S3 — Antigen-specific responses in PBMCs. IL-10, CCL-8, CXCL13, CXCL12, CCL1 and CCL21 were highly expressed by PBMCs from active pulmonary tuberculosis patients after stimulation with either H37Rv lysate or ESAT-6 protein. The data show that both H37Rv lysate and ESAT-6 protein stimulated PBMCs to release antigen-specific cytokines/chemokines. Horizontal bars represent median values, boxes represent the interquartile range (25–75%) and whiskers represent the highest and the lowest values. Horizontal lines indicate a statistically significant difference between groups. *p<0.05; **p<0.005; *** p<0.0005. (TIF) [file pone.0044944.s003.tif]
