# Supplementary material for: Different Patterns of Cytokines and Chemokines Combined with IFN-γ Production Reflect Mycobacterium tuberculosis Infection and Disease
Source: PLoS One. 2012 Sep 13;7(9):e44944. doi: 10.1371/journal.pone.0044944 (PMC3441719; doi:10.1371/journal.pone.0044944)
Supplement: Table S1 — Cytokine/chemokine concentrations in different groups. (DOC) [file pone.0044944.s004.doc]

**Table S1. Examined cytokine/chemokine concentration in different groups**

|  | HD | LTB1 | | LTB2 | | TB | | TP | | LC | | | TPF | | CPF |
| --- | --- | --- | --- | --- | --- | --- | --- | --- | --- | --- | --- | --- | --- | --- | --- |
| **IFN-γ** | 3.51±1.86 | 2.92±0.47 | | 4.87±2.55 | | 2.75±1.24 | | 3.57±2.68 | | 74.41±96.28 | | | 3.64±1.79 | | 8.29±16.74 |
| **IL-1RA** | 57.23±72.86 | 22.34±15.74 | | 270.19 ±348.88 | | 80.78±129.28 | | 229.03±456.76 | | 42.73±57.1 | | | 250.81±277.68 | | 214.15±449.4 |
| **IL-1β** | 4.96±4.16 | 4.86±0.14 | | 5.40±0.85 | |  | | 6.9±10.47 | | 5.09±0.26 | | | 11.95±17.79 | | 6.79±7 |
| **IL2** | 20.24±24.91 | 5.8±11.01 | | 40.17±29.61 | | 19.23±26.47 | | 46.67±75.09 | | 11.72±24.27 | | | 1.26±1.15 | | 4.4±11.16 |
| **IL4** | 0.40±0.32 | 0.27±0 | | 0.27±0 | | 0.38±0.43 | | 1.65±2.62 | | 0.27±0 | | | 2.92±2.95 | | 2.17±3.73 |
| **IL5** | 2.06±0.57 | 2.3±0 | | 2.3±0 | | 2.58±2.7 | | 4.06±7.34 | | 1.94±0.81 | | | 250.81±277.68 | | 214.15±449.4 |
| **IL6** | 12.01±8.75 | 16.34±0.63 | | 21.46±4.34 | | 29396±17.02 | | 53.13±65.76 | | 25.19±5.88 | | | 60048.7±52404.3 | | 18226±26564.4 |
| **IL7** | 5.15±7.56 | 2.09±0 | | 0.88±1.11 | | 3.36±5.79 | | 31.60±76.65 | | 7.18±9.06 | | | 3.97±5.25 | | 11.10±24.82 |
| **IL9** | 3.65±6.71 | 40.21±86.28 | | 73.3±118.15 | | 35.11±40.79 | | 15.82±36.59 | | 1.62±0 | | | 3.41±5.06 | | 1.62±0 |
| **IL10** | 3.55±2.3 | 4.25±0.84 | | 4.25±0.35 | | 3.38±2.52 | | 3.1±2.47 | | 7.17±5.67 | | | 12.94±5.43 | | 19.65±10.88 |
| **IL13** | 1.43±0 | 1.43±0 | | 1.43±0 | | 1.45±0.07 | | 4.28±6.8 | | 3.5±3.72 | | | 41.63±28.41 | | 13.28±11.78 |
| **IL12(p70)** | 3.51±1.86 | 2.92±0.47 | | 4.87±2.55 | | 2.75±1.24 | | 3.57±2.68 | | 3.44±1.12 | | | 3.64±1.79 | | 8.29±16.74 |
| **IL15** | 14.78±11.22 | 9.74±5.43 | | 20.27±14.21 | | 14.29±9.52 | | 17.13±18.1 | | 14.88±12.91 | | | 19.49±5.85 | | 31.21±14.62 |
| **IL17** | 1.42±0 | 1.42±0 | | 4.99±4.99 | | 1.42±0 | | 1.42±0 | | 8.22±15.21 | | | 1.42±0 | | 1.42±0 |
| **GM-CSF** | 117.08 ±127.51 | 138.18±111.61 | | 112.47±161.4 | | 79.09±134.36 | | 70.14±105.72 | | 107.23±115.45 | | | 270.84±454.85 | | 1795.62±2376.2 |
| **G-CSF** | 7.84±12.86 | 1.83±0 | | 1.83±0 | | 7.09±7.97 | | 13.79±4.52 | | 1.83±0 | | | 267.29±185.53 | | 68.22±63.43 |
| **TNF-α** | 5.89±4.94 | 4.6±0 | | 4.6±0 | | 82.56±4.12 | | 61.88±137.51 | | 4.6±0 | | | 72.67±101.22 | | 48.71±102.55 |
| **CXCL5/ENA-78** | 773.43 ±523.86 | 1543.62 ±668.31 | | 999.56±511.93 | | 1295.31±1639 | | 570±381 | | 744.26±613.44 | | 68.7±58.02 | | | 27.24±21.18 |
| **CXCL6/GCP-2/LIX** | 145.34 ±65.17 | 288.62±94.33 | | 258.7±178.96 | | 105.92±46.8 | | 152.82±52.31 | | **-** | | 201.41±236.88 | | | 130.35±0 |
| **CXCL8/IL-8** | 61.81 ±114.13 | 50.07±72.11 | | 85.51±185.7 | | 60.52±91.43 | | 24.78±28.56 | | 130.59±175.58 | | 304.27±237.79 | | | 324.08±486.67 |
| **CXCL9/MIG** | 1908.02± 1560.05 | 2326.25± 1982.77 | | 3392.35± 2887.85 | | 4064.36± 2828.03 | | 5192.27± 4243.401 | | 3192.376± 2151.51 | | 57717.6±22037.3 | | | 4667.42± 2732.76 |
| **IP10/CXCL10** | 828.19 ±390.78 | 988.83 ±412.41 | | 1490.8 ±577.68 | | 2642.36± 3052.23 | | 3264.08 ±2319.5 | | 599.6±327.43 | | 24893.96± 16399.78 | | | 2267.16 ±1032.18 |
| **CXCL11/I-TAC** | 588.18 ±359.73 | 626.03±211.71 | | 956.92 ±449.84 | | 746.46±468.38 | | 848.82±316.9 | | **-** | | 1402.84±1165.57 | | | 1.95±0 |
| **CXCL12/SDF-1** | 3058.3 ±2505.04 | 2552.36 ±571.13 | | 3455.73 ±919.35 | | 2884.58± 1375.55 | | 3117.2±1828.65 | | 2341.49 ±1525.28 | | 11767.09±6084.23 | | | 4699.12± 2391.44 |
| **CXCL13/BCA-1** | 28.49±10.53 | 57.52±53.31 | | 41.91±20.51 | | 38.74±30.88 | | 38.97±16.11 | | 31.14±38.56 | | 143.97±89.77 | | | 302.64±353.85 |
| **CCL1/I-309** | 1.18±0.75 | 1.0±0.56 | | 2.17±2.47 | | 3.05±2.53 | | 3.92±3.33 | | 1.83±1.46 | | 63.76±36.9 | | | 5.19±5.57 |
| **CCL2/MCP-1** | 330.58 ±215.1 | 170.3±148.39 | | 159.89 ±141.19 | | 208.43±143.9 | | 346.17±126.14 | | 471.51±134.55 | | 3408.5±2930.09 | | | 2111.73±2257.72 |
| **CCL3/MIP-1a** | 104.55 ±50.05 | 99.19±18.95 | | 102.76±47.37 | | 142.63±145.29 | | 79.16±23.14 | | **-** | | 166.98±124.75 | | | **-** |
| **CCL4/MIP-1b** | 92.98 ±113.34 | 96.06±64.01 | | 145.33 ±243.96 | | 58.69±36.4 | | 56.69±55.15 | | **-** | | 175.96±274.1 | | | 33.28±0 |
| **CCL5/RANTES** | 31512.35± 49660.17 | 12247.24± 15189.9 | | 26451.5± 51415.45 | | 8215.69± 10452.01 | | 5500.12 ±2112.72 | | 7437.14 ±5422.83 | | 187.92±143.74 | | | 82.47±202.83 |
| **CCL7/MCP-3** | 419.26 ±878.89 | 635.24 ±1018.93 | 635.5 ±1020.53 | | 207.36±648.02 | | 207.36±648.02 | | **-** | | 1788.7±1021.16 | | | 2277.59±0 | |
| **CCL8/MCP-2** | 31.21±11.81 | 31.17±7.21 | 28.63±14.9 | | 26.54±10.32 | | 30.12±22.55 | | 66.72±29.85 | | 1090.803±924.81 | | | 83.48±114.56 | |
| **CCL11/Eotaxin** | 285.46 ±137.68 | 390.54 ±163.25 | 350.75 ±142.78 | | 255.19±130.16 | | 330.14±185.56 | | 72.46±56.64 | | 136.95±136.11 | | | 46.84±47.51 | |
| **CCL13/MCP-4** | 67.6±45.48 | 68.9±43.55 | 66.43±52.64 | | 51.64±39.86 | | 51.95±35.93 | | **-** | | 36.89±27.33 | | | 78.33±110.21 | |
| **CCL15/MIP-1d** | 3480.46 ±2419.69 | 3313.48 ±2875.0 | 3895.58 ±2282.9 | | 4037.31±3750.0 | | 5477.17±6183.4 | | **-** | | 2264.11±2297.24 | | | 2433.547±0 | |
| **CCL17/TARC** | 99.94±67.16 | 78.67±20.91 | 110.95±106.14 | | 79.27±80.2 | | 65.18±42.01 | | **-** | | 82.9±103.88 | | | 6.87±0 | |
| **CCL20/MIP-3a** | 22.8±7.56 | 21.33±5 | 21.09±9.52 | | 23.49±7.92 | | 20.77±3.6 | | **-** | | 292.34±578.7 | | | 49.51±0 | |
| **CCL21** | 160.98 ±113.62 | 170.98±94.55 | 218.21±107.7 | | 191.96±134.2 | | 188.26±116.06 | | 188.71±108.48 | | 3878.91±3581.91 | | | 790.21±725.86 | |
| **CCL24/Eotaxin-2** | 243.91 ±123.1 | 272.89 ±227.78 | 292.55 ±220.48 | | 242.46±211.52 | | 224.41±248.34 | | **-** | | 315.2±203.55 | | | 224.92±0 | |
| **CCL26/Eotaxin-3** | 58.69±45.71 | 45.66±38.92 | 58.67±29.58 | | 41.64±23.68 | | 52.82±47.09 | | **-** | | 125.33±129.05 | | | 48.8±0 | |
| **CCL27/CTACK** | 617.55 ±200.76 | 619.3±269.29 | 567.09 ±145.26 | | 607.11±222.71 | | 714.51±409.2 | | 840.63±455.37 | | 321.32±132.3 | | | 330.09±123.55 | |
